# Increased NQO1 but Not c-MET and Survivin Expression in Non-Small Cell Lung Carcinoma with *KRAS* Mutations

**DOI:** 10.3390/ijerph110909491

**Published:** 2014-09-12

**Authors:** Ahmet Yilmaz, Nehad Mohamed, Kara A. Patterson, Yan Tang, Konstantin Shilo, Miguel A. Villalona-Calero, Michael E. Davis, Xiaoping Zhou, Wendy Frankel, Gregory A. Otterson, Howard D. Beall, Weiqiang Zhao

**Affiliations:** 1 Department of Pathology, The Ohio State University, Columbus, OH 43210, USA; E-Mails: Ahmet.Yilmaz2@osumc.edu (A.Y.); Nehad.Mohamed@osumc.edu (N.M.); Kara.Patterson@osumc.edu (K.A.P); Yan.Tang@osumc.edu (Y.T.); Konstantin.Shilo@osumc.edu (K.S.); Xiao-Ping.Zhou@osumc.edu (X.Z.); Wendy.Frankel@osumc.edu (W.F.); 2 Department of Internal Medicine, The Ohio State University, Columbus, OH 43210, USA; E-Mails: Miguel.Villalona@osumc.edu (M.A.V.-C.); Greg.Otterson@osumc.edu (G.A.O.); 3 Department of Animal Sciences, The Ohio State University, Columbus, OH 43210, USA; E-Mail: Davis.28@osu.edu; 4 Department of Pharmaceutical Sciences, School of Pharmacy, The University of Montana, Missoula, MT 59812, USA; E-Mail: Howard.Beall@mso.umt.edu

**Keywords:** lung cancer, non-small cell lung carcinoma, oxidative stress, KRAS, mutation, NQO1, DNA methyl transferase, ERK1/2, c-MET, survivin

## Abstract

Cigarette smoking is one of the most significant public health issues and the most common environmental cause of preventable cancer deaths worldwide. EGFR (Epidermal Growth Factor Receptor)-targeted therapy has been used in the treatment of LC (lung cancer), mainly caused by the carcinogens in cigarette smoke, with variable success. Presence of mutations in the *KRAS* (Kirsten rat sarcoma viral oncogene homolog) driver oncogene may confer worse prognosis and resistance to treatment for reasons not fully understood. NQO1 (NAD(P)H:quinone oxidoreductase), also known as DT-diaphorase, is a major regulator of oxidative stress and activator of mitomycins, compounds that have been targeted in over 600 pre-clinical trials for treatment of LC. We sequenced *KRAS* and investigated expression of NQO1 and five clinically relevant proteins (DNMT1, DNMT3a, ERK1/2, c-MET, and survivin) in 108 patients with non-small cell lung carcinoma (NSCLC). NQO1, ERK1/2, DNMT1, and DNMT3a but not c-MET and survivin expression was significantly more frequent in patients with *KRAS* mutations than those without, suggesting the following: (1) oxidative stress may play an important role in the pathogenesis, worse prognosis, and resistance to treatment reported in NSCLC patients with *KRAS* mutations, (2) selecting patients based on their *KRAS* mutational status for future clinical trials may increase success rate, and (3) since oxidation of nucleotides also specifically induces transversion mutations, the high rate of *KRAS* transversions in lung cancer patients may partly be due to the increased oxidative stress in addition to the known carcinogens in cigarette smoke.

## 1. Introduction

Cigarette smoking, the main cause of lung cancer, has remained as one of the most significant public health issues [1,2]. Lung cancer is the most frequent cause of cancer deaths worldwide [3]. It comprises approximately 18.2% of all cancer deaths, causing nearly as many deaths as breast, prostate, and colon cancers combined [3]. Although EGFR-directed therapy has been used for treatment with variable success, it may be less effective in patients carrying mutations in *KRAS* [4].

**Figure 1:**
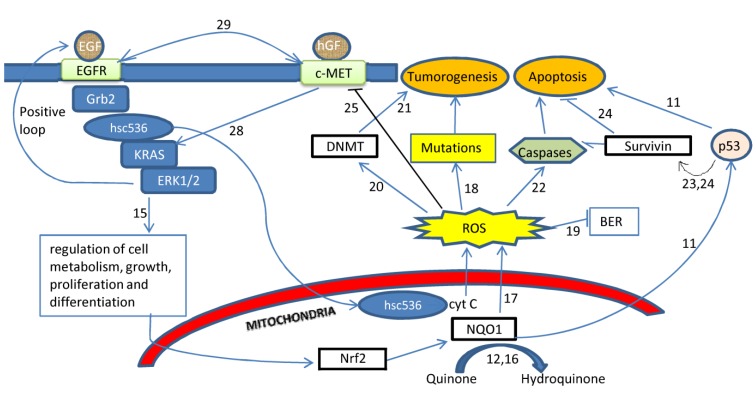
Inter-relationships among oxidative stress, *KRAS*, NQO1, survivin, DNA methyltransferases, and c-MET in the pathogenesis of lung cancer ^a,b^.

^a^ See the list of references for literature cited. ^b^ Activating mutations in *KRAS* results in increased cellular growth, metabolism, and proliferation [15]. The reactive oxygen species (ROS) produced during metabolic processes are normally converted into harmless products by antioxidant enzymes such as NQO1 [12,16] which stabilizes the tumor suppressor TP53 [11]. However, when the capacity of NQO1 and other antioxidant enzymes is overwhelmed, the ROS may “leak out” of the mitochondria [17] and oxidize DNA bases, creating mutations [18]. Oxidative stress may repress base excision repair systems, which may result in additional mutations [19]. ROS may increase DNA methyl transferase activity [20], which may be an early event in lung carcinogenesis [21]. ROS regulate apoptosis via upregulation of caspases [22]. Survivin, a caspase inhibitor, also regulated by DNMTs and TP53, may decrease effects of ROS by inhibiting apoptosis [23,24]. Oxidative stress inhibits expression of c-MET, an oncogene altered in many cancers [25,26,27]. c-MET regulates oncogenic transformation of the lung cancers associated with *KRAS* mutations [28]. Co-activation of c-MET and EGFR is seen in a subset of lung cancers with distinct expression profile, mutational spectrum, and response to chemotherapy [29].

*KRAS* is a driver oncogene encoding for a small GTPase [5]. It activates proteins such as RAF, MEK, and ERK1/2 involved in the MAPK/ERK signal transduction pathway in response to extracellular signals received by the EGFR [6], (Figure 1). Mutations in *KRAS* result in the loss of its GTPase activity and constitutive activation of the downstream proteins, resulting in malignant transformation [7].

An interesting feature of the *KRAS* mutations in smokers is the high incidence of G:C > A:T transversions [8]. Previous studies have shown that NNK (4-(*N*-Methyl-*N*-nitrosamino)-1-(3-pyridyl)-1-butanone), the carcinogen found in cigarette smoke, is one of the causes of these transversion mutations [9]. Interestingly, DNA replication involving 8-OHdG, the product of oxidation of guanosine, also produces the G:C > A:T transversion mutations [10]. Increased transversion rates in smokers, thus, may not only be due to the NNK but also increased oxidative stress (OS) in lung cancer cells, suggesting that there may be a link between oxidative stress and *KRAS* mutational status.

NQO1 (NAD(P)H:quinone oxidoreductase, also known as DT-diaphorase) is a major regulator of oxidative stress that links oxidative stress and tumorogenesis by stabilizing the tumor suppressor TP53 [11]. Its overexpression in the tumor but not normal tissue has made it an attractive target for treatment of lung cancer [12]. NQO1 is the main activator of quinone-containing alkylating agents such as mitomycins.

To our knowledge, possible associations between NQO1 expression and *KRAS* mutational status have been rarely investigated in the literature. As of February 2014, entering “KRAS” and “NQO1” into the PubMed database [13] returned only one relevant study that investigated modulation of *RAS* (rat sarcoma viral oncogene homolog) mutations by NQO1 [14]. Our objective was to sequence *KRAS* and compare expression of NQO1 as well as a panel of five clinically relevant proteins in 108 non-small cell lung carcinoma (NSCLC), the most common form of LC, patients with and without *KRAS* mutations. The panel included survivin (a potent inhibitor of apoptosis), DNMT1 (the maintenance methylator of DNA), DNMT3a (the enzyme ensuring accurate inheritance of the maternal methylation patterns), ERK1/2 (downstream targets of *KRAS*), and c-MET (an oncogene important in the transformation of cells with *KRAS* mutations).

## 2. Results

In total, 52 of the 108 (48.1%) patients included in this study had mutations in *KRAS*. Of the mutations, 73.1% (38/52) were transversions and the remaining were transitions. All of the mutations originated from the guanosine nucleotide.

Average age at diagnosis was 61.0 (8.9) years (Supplemental Table S1). Male gender, smoking history, hypertension, and family cancer history were present in over half of all patients. Smoking history was present in 44 of the 48 patients (91.7%) with *KRAS* mutations.

Immunohistochemistry (IHC) results showed that NQO1, DNMT1, DNMT3a, and ERK1/2 but not survivin and c-MET expression were more frequent in patients carrying mutations in *KRAS* than those carrying the wild type (Table 1). When the *KRAS* mutational status was ignored, expression of the following proteins was present in over half of the NSCLC patients (percentage of patients with positive expression of the protein in parenthesis): NQO1 (67.8%), survivin (75%), DNMT1 (74.6%), and c-MET (68.9%). DNMT3a and ERK1/2 expression, on the other hand, were detected in less than half of the patients (*i.e.*, 43.3% and 46.2%, respectively).

**Table 1:** Expression of NQO1 and clinically relevant proteins in non-small cell lung carcinoma patients with and without *KRAS* mutations.

Protein	IHC ^a^	*KRAS* Wild Type ^b^	*KRAS* Mutated ^b^	Total	*P* ^c^
NQO1	Negative	17	2	19	<0.001 *
	Positive	16	24	40	
DNMT1	Negative	14	1	15	<0.001 *
	Positive	19	25	44	
DNMT3a	Negative	24	10	34	0.01 *
	Positive	10	16	26	
ERK1/2	Negative	17	11	28	0.002 *
	Positive	4	20	24	
c-MET	Negative	5	9	14	0.43
	Positive	15	16	31	
Survivin	Negative	3	10	13	0.19
	Positive	19	20	39	

^a^ IHC = results of the immunohistochemical staining. ^b^ Values in these columns represent numbers of patients with positive or negative immunohistochemical staining. ^c^ An asterisk “*” denotes statistical significance at *P* = 0.05.

**Figure 2:**
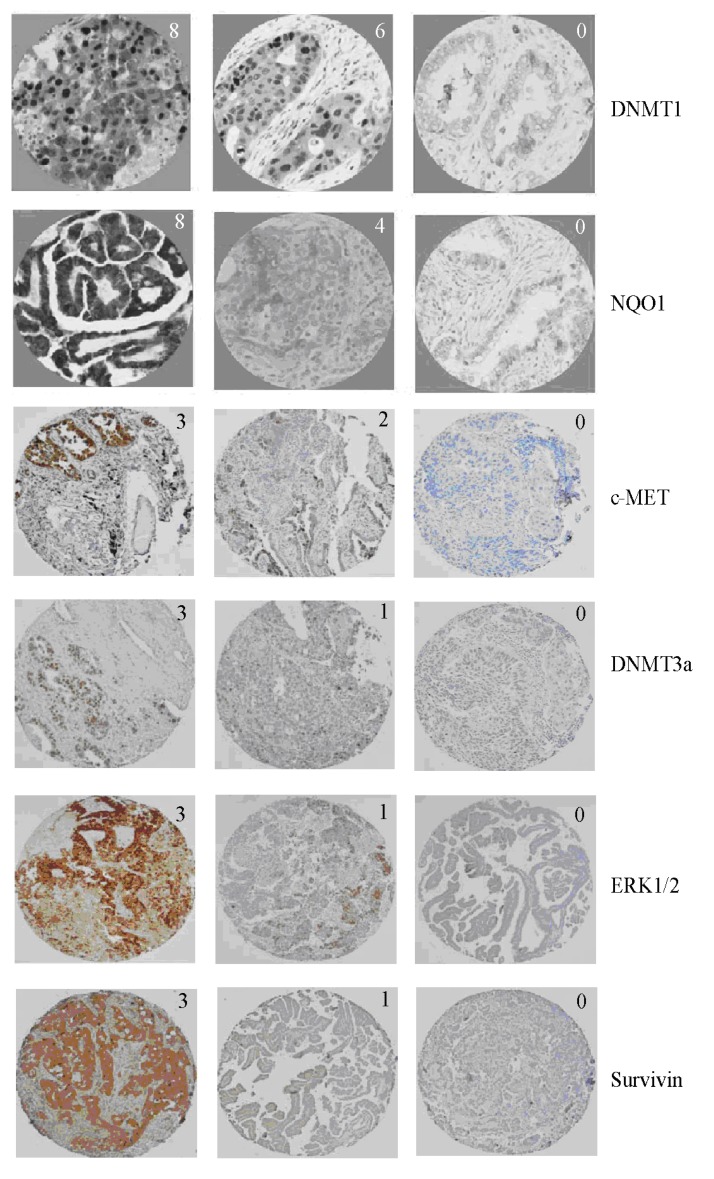
Representative immunohistochemistry images of proteins with different Allred scores investigated in this study. The values on the top right corner of each image represent Allred score for that image.

## 3. Discussion

Despite all efforts, lung cancer has remained as the leading cause of cancer deaths worldwide [30,31]. The median survival rate after diagnosis of advanced-stage lung cancer is approximately 7–8 months and the average one year survival rate can be as low as 32% [32]. The reasons for these high mortality rates are probably the essential functions of the lungs, lack of reliable methods for early detection and prevention of the cancer, and the unavailability of optimized therapeutic options. Although EGFR-targeted therapy has been used with variable success, it may be less effective in patients carrying mutations in *KRAS* for reasons not fully understood [4,33].

*KRAS* is a small GTPase involved in the MAPK/ERK signal transduction pathway that regulates cell proliferation, differentiation and senescence [34,35]. Activating mutations in *KRAS* result in the constitutive activation of the downstream proteins and tumorogenesis [36]. *KRAS* mutations have been widely reported in many cancers including lung and colorectal cancers [37].

Evidence in the literature suggests that oxidative stress and *KRAS* mutational status may be related. The vast majority of the *KRAS* mutations reported in lung cancer patients are transversions. Interestingly, increased oxidative stress is also associated with increased transversion rates. Oxidative stress suppresses base excision repair (BER) mechanisms [19], which may result in incorporation of oxidized DNA nucleotides, especially guanosine, into the replicating DNA, generating transversions. We suggest that a two way relationship that may exist between the *KRAS* mutational status and oxidative stress may be the starting point for initiating a vicious cycle leading to malignant transformation in NSCLC.

NQO1 is an especially interesting antioxidant enzyme that may represent a direct link between oxidative stress and tumorogenesis. It not only catalyzes the two electron reduction of quinones into the hydroquinones in a reaction that prevents the production of harmful semiquinones and ROS [16,38] but also stabilizes the tumor suppressor TP53 [11]. The C609T polymorphisms in NQO1 may be a predictive factor for survival after resection of NSCLC tumors [39] and the rs1800566C/T SNP within NQO1 is linked to the deletion of EGFR exon 19 in NSCLC patients [40]. Eighty-four percent of the NQO1(-/-), but none of the control mice, exposed to gamma irradiation develop NSCLC [41].

The catalytic function of NQO1 in the reduction of quinones to hydroquinones is required for the activation of quinone-containing alkylating agents such as mitomycins, an important group of compounds used in the treatment of lung cancer for decades [12]. NQO1 expressed by the tumor cells activates the quinone-containing alkylating agents which results in the death of the cells that express NQO1. This phenomenon has been exploited to the extreme in the search for compounds for effective treatment of LC. As of 2013, over 600 pre-clinical trials targeting mitomycins have been performed but the success rate has been disappointingly low. Mitomycin C has remained as the only quinone-containing alkylating agent approved by the Food and Drug Administration for lung cancer treatment. Despite the overwhelming evidence suggesting importance of both NQO1 and *KRAS* in the pathogenesis of cancers, we are aware of only a single study investigating possible associations between them [14].

In our study, positive NQO1 staining was significantly more frequent in NSCLC patients with mutated than with wild type *KRAS* (92% *vs.* 48%, *P* <0.001). This observation raises some interesting questions. For example, why is there a stronger need for detoxification of quinones and stabilization of TP53 in NSCLC cells with the *KRAS* mutations than in those carrying the wild type? More importantly, could the increased NQO1 have any direct involvement in the worse prognosis and resistance to treatment seen in NSCLC patients with *KRAS* mutations? Further investigations are required to find definitive answers to these questions. However, the significantly more frequent expression of NQO1 in the NSCLC with *KRAS* mutations found in our study and the wide range of cellular events regulated by oxidative stress imply an important role for oxidative stress in the development of NSCLC with the *KRAS* mutations.

Another interesting result obtained in our study was the increased expression of DNMT1, the enzyme that methylates promoters of tumor suppressor genes in cancer cells, in NSCLC with *KRAS* mutations. Positive DNMT1 staining was seen in 57.6% of patients carrying the wild type but 96.2% of the patients with the mutated *KRAS*. Positive staining for DNMT1 but negative staining for DNMT3a was most frequent in NSCLC patients when the *KRAS* mutational status was ignored. These results are in indirect agreement with a previous study [42].

In our study, ERK1/2 expression was elevated in the NSCLC with *KRAS* mutations but survivin and c-MET expression did not differ between the patients with and without *KRAS* mutations. Survivin inhibits apoptosis by repressing caspase activity and regulating ROS production [43,44,45,46]. Its presence in tumor but not terminally differentiated cells makes it an attractive candidate for chemotherapy [47,48] and clinical trials for its therapeutic use in lung cancer are ongoing [49]. The lack of an association between *KRAS* mutational status and survivin expression in our study suggests that survivin-mediated apoptosis is probably not an important event in the development of the phenotype associated with *KRAS* mutations. When *KRAS* mutational status was ignored, survivin was expressed in 75% of all NSCLC tumors included in this study, a result in agreement with a previous study reporting overexpression of survivin in NSCLC [50].

In summary, we sequenced *KRAS* in NSCLC patients and investigated the expression of several proteins involved in oxidative stress and related events in an attempt to determine the molecular changes associated with the *KRAS* mutations which are known to confer worse prognosis and less favorable response to therapy. Our results suggest that increased expression of NQO1, and consequently oxidative stress, may play an important role in the development of NSCLC with *KRAS* mutations. Further investigations of the components of the oxidative stress systems may help in the efforts aimed at identifying molecular targets for the treatment of NSCLC with *KRAS* mutations. Contribution of survivin-mediated apoptosis and c-MET expression in the development of NSCLC, on the other hand, is probably not significant although further research is required to substantiate this conclusion. These results may help in a better understanding of the effects of *KRAS* mutations in not only NSCLC but also other types of cancers such as colorectal cancer where *KRAS* mutational status is a major determinant in the selection of optimal therapeutic regimes for individual patients.

## 4. Experimental Section

The samples included in this study were obtained from 108 NSCLC patients treated at the Ohio State University Wexner Medical Center in Columbus, OH, USA. DNA was isolated using QiaAmp Micro DNA kits from Qiagen Inc. (Valencia, CA, USA). The tissue samples resected after surgery were embedded in paraffin, fixed in formalin, and archived until analyzed. We used a semi-nested Polymerase Chain Reaction (PCR) assay followed by direct sequencing to identify KRAS mutations in codons 12 and 13. The forward 5’-TACTGGTGGAGTATTTGATAGTG-3’ and reverse 5’-CTGTATCAAAGAATGGTCCTG-3’ primers were used in the first round of PCR. In the second round of PCR, the forward 5’-TGTAAAACGGCCAGTTAGTGTATTAACCTTATGTG-3’ and reverse 5’-CAGGAAACAGCTATGACCACCTCTATTGTTGGATCATATTCG-3’ primers were used. The PCR conditions (95 °C for 15 min, 30 cycles of 94 °C for 30 s, 48 °C for 30 s, and 72 ° C for 30 s, followed by an extension step of seven minutes at 72 °C) were used in both rounds of PCR except that the annealing temperature was raised to 58 °C in the second round. Fluorescence-based capillary electrophoresis was used in an ABI3130XL genetic analyzer to detect the mutations.

To perform IHC, tissue blocks were cut at four micron thickness and placed on positively charged slides. Slides with sections were placed in a 60 °C oven for one hour and then cooled. The samples were deparaffinized and rehydrated through two changes of O-xylene for 5 min each and 10–20 dips in graded ethanol solutions. The slides were quenched for five minutes in 3% H_2_O_2_ solution in water to block endogenous peroxidases. To perform antigen retrieval, the slides were placed in Target Retrieval Solution (Dako, CA, USA) for 25 min at 96 °C in a vegetable steamer (Black & Decker, IL, USA) and cooled for 15 min in solution. Slides were processed using a Dako Autostainer Immunostaining System following manufacturer’s instructions. Initially, slides were blocked with serum-free protein (Dako) for 15 min. The primary antibodies were diluted at proportions given in Supplemental Table S2 and incubated for one hour. Detection systems described in Supplemental Table S2 were used. Staining was visualized after incubation with DAB+ chromogen (Dako) for five minutes. The slides were then counterstained in hematoxylin, dehydrated through graded ethanol solutions and coverslipped. Representative IHC images are provided in Figure 2.

IHC scores were obtained using Allred scoring system which combines the percentage of positive cells and the intensity of the reaction products in the carcinoma [51]. Proportion score has six possible values based on percentage of positive cells (in parentheses): 0 (0%), 1 (<1%), 2 (1%–10%),3 (11%–33%), 4 (34%–66%), and 5 (>67%). Intensity score has four possible values (with level of intensity in parentheses): 0 (none), 1 (weak), 2 (intermediate), and 3 (strong). The proportion and intensity scores are added to obtain the final Allred scores that range from 0 to 8. We reported samples with the final Allred scores 0, 1, and 2 as negative and those with scores 3 or higher as positive. Statistical analysis was performed using the chi-square or Fisher’s exact test for categorical variables and the F-test for continuous variables with the significance level set at *P* = 0.05.

## 5. Conclusions

Based on the results obtained in this study, we conclude the following: (1) increased NQO1 expression in NSCLC patients with KRAS mutations indicates that cells carrying *KRAS* mutations may be suffering from increased oxidative stress, (2) the increased transversion rates in NSCLC patients with *KRAS* mutations may partly be due to increased oxidative stress that induces transversions, and (3) since the patients with mutations may utilize quinone-containing alkylating agents more efficiently due to increased NQO1 expression, including *KRAS* mutational status in future clinical trials may improve success rates.

## Supplementary Files

Supplementary File 1
