# Supplementary material for: Increased NQO1 but Not c-MET and Survivin Expression in Non-Small Cell Lung Carcinoma with KRAS Mutations
Source: Int J Environ Res Public Health. 2014 Sep 12;11(9):9491–502. doi: 10.3390/ijerph110909491 (PMC4199031; doi:10.3390/ijerph110909491)
Supplement: Supplementary File 1 [file ijerph-11-09491-s001.docx]

Supplementary Information

Increased NQO1 but Not c-MET and Survivin Expression in
Non-Small Cell Lung Carcinoma with *KRAS* Mutations

**Table S1.** Clinical parameters in non-small cell lung cancer patients with *KRAS* mutations.

| **Variables** | **Patients (%) ^a^** |
| --- | --- |
| Patients | 52 |
| Average age (SD), years | 61.0 (8.9) |
| Male gender | 26/51 (51.0) |
| Smoker | 44/48 (91.7) |
| Consumes alcohol | 12/47 (25.5) |
| Diabetic | 8/47 (17.0) |
| Dyslipidemia present | 20/47 (42.6) |
| Hypertension present | 32/48 (66.7) |
| Individual cancer history present | 6/47 (12.8) |
| Family cancer history present | 33/47 (70.2) |

^a^ Number of patients with each variable. Values in parentheses represent percentages.

**Table S2.** Details of the immunohistochemistry protocols used to investigate expression of proteins included in this study.

| **Protein** | **Host of the Primary Antibody** | **Supplier of the Primary Antibody** | **Catalog Number** | **Dilution Factor** | **Detection Method ^a,b,c,d^** |
| --- | --- | --- | --- | --- | --- |
| NQO1 | Mouse monoclonal | Abcam | Ab28947 | 1:700 | EDL |
| DNMT1 | Rabbit polyclonal | Abcam | Ab19905 | 1:250 | BPR |
| DNMT3a | Rabbit polyclonal | Santa Cruz Biotech. | SC20703 | 1:250 | GAR |
| ERK1/2 | Rabbit monoclonal | Cell Signaling Tech. | 4370S | 1:100 | GAR |
| c-MET | Rabbit polyclonal | Santa Cruz Biotech. | SC-10 | 1:250 | GAR |
| Survivin | Mouse monoclonal | Santa Cruz Biotech. | SC-17779 | 1:100 | Mach 3 |

^a^ EDL = EnVision Plus Dual Link System-HRP (Dako), a labeled polymer detection system, was applied for
30 min. ^b^ BPR = Bond Polymer Refine Detection system (Leica) employing Poly-HRP IgG was used following manufacturer’s recommendations except that DAB+ chromogen (Dako) was applied for 10 min. ^c^ GAR = Goat anti-rabbit secondary antibody (Vector Laboratories, catalog number BA-1000) diluted 1:200 in 2% normal goat serum was added on the slide and incubated for 20 minutes. Vectastain Elite Standard ABC kit-HRP detection system was applied for 30 min. ^d^ Mach 3 = Mach 3 Mouse Probe and Mach 3 Mouse Polymer-HRP (Biocare) were applied sequentially for 20 min each.

© 2014 by the authors; licensee MDPI, Basel, Switzerland. This article is an open access article distributed under the terms and conditions of the Creative Commons Attribution license (http://creativecommons.org/licenses/by/3.0/).
